# Differential cellular and humoral immune responses in immunocompromised individuals following multiple SARS-CoV-2 vaccinations

**DOI:** 10.3389/fcimb.2023.1207313

**Published:** 2023-06-23

**Authors:** Rhys T. Meredith, Max D. Bermingham, Kirsten Bentley, Sayeh Agah, Abigail Aboagye-Odei, Ross A. R. Yarham, Hayley Mills, Muddassir Shaikh, Neil Hoye, Richard J. Stanton, David R. Chadwick, Maria A. Oliver

**Affiliations:** ^1^ InBio, Cardiff, United Kingdom; ^2^ Division of Infection and Immunity, School of Medicine, Cardiff University, Cardiff, United Kingdom; ^3^ InBio, Charlottesville, VA, United States; ^4^ Department of Infectious Diseases, South Tees Hospitals National Health Service (NHS) Foundation Trust, Middlesbrough, England, United Kingdom; ^5^ Department of Kidney Services, South Tees Hospitals National Health Service (NHS) Foundation Trust, Middlesbrough, England, United Kingdom; ^6^ Department of Rheumatology, South Tees Hospitals National Health Service (NHS) Foundation Trust, Middlesbrough, England, United Kingdom

**Keywords:** SARS-CoV-2, vaccine efficacy, immunocompromised cohorts, T cell responses, antibody production, third/fourth doses

## Abstract

**Introduction:**

The heterogeneity of the immunocompromised population means some individuals may exhibit variable, weak or reduced vaccine-induced immune responses, leaving them poorly protected from COVID-19 disease despite receiving multiple SARS-CoV-2 vaccinations. There is conflicting data on the immunogenicity elicited by multiple vaccinations in immunocompromised groups. The aim of this study was to measure both humoral and cellular vaccine-induced immunity in several immunocompromised cohorts and to compare them to immunocompetent controls.

**Methods:**

Cytokine release in peptide-stimulated whole blood, and neutralising antibody and baseline SARS-CoV-2 spike-specific IgG levels in plasma were measured in rheumatology patients (n=29), renal transplant recipients (n=46), people living with HIV (PLWH) (n=27) and immunocompetent participants (n=64) post third or fourth vaccination from just one blood sample. Cytokines were measured by ELISA and multiplex array. Neutralising antibody levels in plasma were determined by a 50% neutralising antibody titre assay and SARS-CoV-2 spike specific IgG levels were quantified by ELISA.

**Results:**

In infection negative donors, IFN-γ, IL-2 and neutralising antibody levels were significantly reduced in rheumatology patients (p=0.0014, p=0.0415, p=0.0319, respectively) and renal transplant recipients (p<0.0001, p=0.0005, p<0.0001, respectively) compared to immunocompetent controls, with IgG antibody responses similarly affected. Conversely, cellular and humoral immune responses were not impaired in PLWH, or between individuals from all groups with previous SARS-CoV-2 infections.

**Discussion:**

These results suggest that specific subgroups within immunocompromised cohorts could benefit from distinct, personalised immunisation or treatment strategies. Identification of vaccine non-responders could be critical to protect those most at risk.

## Introduction

COVID-19 vaccination programmes have been under way for more than two years and have successfully prevented tens of millions of deaths globally. In the first year, it was estimated that 19.8 million deaths from COVID-19 were averted by vaccination with SARS-CoV-2 (Watson et al., 2022). Despite this success, there is a substantial number of individuals who do not respond well to SARS-CoV-2 vaccinations. Variable, weak, or reduced vaccine immune responses have been observed in immunocompromised people compared to immunocompetent individuals (Nadesalingam et al., 2022; Parker et al., 2022; Lu et al., 2023). Consequently, immunocompromised individuals have been shown to have a greater risk of severe or persistent SARS-CoV-2 viral infection, with persistent infection favouring the emergence of newer variants (Abbasi, 2021; Belsky et al., 2021; Agrawal et al., 2022; Freer and Mudaly, 2022; Mishra et al., 2022). With immunocompromised cohorts excluded from initial vaccine trials, data on the effectiveness of the vaccines in these groups was initially lacking. Since vaccination began in December 2020, data on vaccine efficacy and safety in these groups has been acquired. Meta-analyses found that in immunocompromised patients (solid organ transplant recipients (SOTR), solid and haematological cancer patients, patients with immune mediated inflammatory disorders, malignant diseases, and inflammatory rheumatic disease), seroconversion rates and SARS-CoV-2 specific immunoglobulin G (IgG) antibody levels were considerably lower than in the control groups, with organ transplant recipients showing the lowest rates of seroconversion (Kolb et al., 2021; Rincon-Arevalo et al., 2021; Lee et al., 2022; Marra et al., 2022; Smith et al., 2022). Patients with primary immunodeficiencies, patients with rheumatological conditions and kidney transplant recipients were found to have much lower cellular responses after two vaccinations than their healthy control counterparts (Stumpf et al., 2021; Oyaert et al., 2022; Reischig et al., 2022). Not all studies agree. One study concluded that although there was a reduction in seroconversion in patients with rheumatoid arthritis and seronegative spondyloarthritis after the first vaccine, by the second, 100% of patients studied had seroconverted (Simader et al., 2022). Data on vaccine efficacy in people living with HIV (PLWH) has been mostly encouraging, with several studies suggesting that PLWH that are on ART have comparable cellular and humoral vaccine-induced immune responses to healthy control groups following two doses of COVID-19 vaccines (Frater et al., 2021; Oyaert et al., 2022), although PLWH with higher viral loads and/or low CD4 cell counts may have lower seroconversion and IgG levels (Xu et al., 2022). Contradictory data suggests that vaccination in PLWH results in a robust T cell response but an inferior neutralising antibody response (Lv et al., 2022).

Despite the conflicting findings and disparity in results between different cohorts of immunocompromised individuals, many countries advise all immunocompromised individuals to receive additional booster vaccine doses. In PLWH, a third dose was found to increase the humoral and cellular response above that measured after two doses, with responses comparable to healthy controls (Tortellini et al., 2022). In SOTR, a third dose was found to increase SARS-CoV-2 specific IgGs and neutralising antibodies, although responses were still comparatively weak compared to healthy controls (Ferreira et al., 2022; Karaba et al., 2022; Peghin et al., 2022). In rheumatology patients, conflicting data has been reported, with some studies suggesting a third dose results in excellent seroconversion of nearly all patients (Azzolini et al., 2022; Isnardi et al., 2022), some suggesting that only cellular immunity is boosted (Jyssum et al., 2022), and some suggesting that cellular and humoral responses are increased but at a much lower level than healthy controls (Farroni et al., 2022). Given the conflicting data on booster vaccines so far, the extent to which protection from additional vaccines is enhanced in immunocompromised groups remains unclear (Nadesalingam et al., 2022; Parker et al., 2022).

Here, we recruited immunocompetent participants (control group) and individuals from three different immunocompromised cohorts: rheumatology patients, renal transplant recipients, and PLWH, who had received at least three SARS-CoV-2 vaccine doses. Using a rapid whole-blood assay to measure T cell cytokine release (Murugesan et al., 2020; Petrone et al., 2021; Tan et al., 2021; Oliver et al., 2022), a neutralising antibody assay (Fielding et al., 2022) and an enzyme-linked immunosorbent assay (ELISA) to measure total IgG, we compared their immune responses to an immunocompetent control group. The aim was to investigate the relationship between multiple SARS-CoV-2 vaccinations and both cellular and humoral immune responses of at-risk immunocompromised individuals. Disparity in results may exist between previous studies due to limited parameters being used to assess immunity. To address this issue and add clarity to the field, our study measured IFN-γ production, IL-2 production, baseline IgG levels and neutralising antibody levels, all from the same sample of blood to build a more complete picture of immunity in multiple immunocompromised cohorts following three or four vaccines. Comparing responses between multiple immunocompromised cohorts with each other as well as with immunocompetent controls provides informative data about which groups of individuals are most at risk.

## Materials and methods

### Study approval

This study received ethical approval from the Wales Research Ethics Committee (REC) 6 (IRAS number: 305040). Written informed consent was received from participants prior to inclusion in the study.

### Study cohort

This study received ethical approval from the Wales Research Ethics Committee (REC) 6 (IRAS number: 305040). Participants from across Wales and England were recruited to the project between December 2021 and May 2022. Immunocompromised patients were recruited when attending clinics and blood samples taken to coincide with routine blood tests. Participants were only recruited if they met the eligibility criteria stated in documents supplied to the REC, namely be aged 18 years of older, be able to understand and communicate in English or in first language with translator, have had at least 3 doses of a UK approved SARS-CoV-2 vaccine, and fall into one of the four cohorts of patients being investigated: rheumatology patients established on biologic therapy, renal transplant patients taking immunosuppressants, PLWH, and immunocompetent (control) group. All immunocompromised participants were identified via National Health Service (NHS) clinic and directorate databases and patients were all diagnosed by NHS professionals, with their underlying diagnoses recorded where appropriate (see **Supplemental Tables S1, S2**). All participants had to be able and willing to provide informed, written consent prior to inclusion. Individuals were not eligible for enrolment into the study if they met any of the exclusion criteria, namely they were unable to provide consent, had a predicted life expectancy less than one year, had been in receipt of rituximab in the past year, or had been in receipt of intravenous immunoglobulin (IVIG) therapy in the past 3 years. For the rheumatology patients, exclusion criteria also included receiving anti-TNF or other biologic therapies <3 months. For the renal transplant recipients, exclusion criteria also included having been on maintenance immunotherapy other than tacrolimus, mycophenolate-mofetil and prednisolone; or having been on these treatments for < 3 months. For the PLWH cohort, exclusion criteria also included being on antiretroviral therapy for <2 years; HIV viral load >100 copies/ml at any point in past 18 months; any opportunistic infection or diagnosis of AIDS-related malignancy in the past 6 months. For the immunocompetent group, exclusion criteria also included having any chronic diseases or medical conditions affecting the immune system or taking any medication which impairs the immune system and thus deems them as immunocompromised in some way. A total of 166 participants met the inclusion criteria, each of whom donated a single sample of blood. At the time of blood sample collection, corresponding details of prior test results for SARS-CoV-2 infection including method of confirmation (polymerase chain reaction test and/or lateral flow), details of SARS-CoV-2 vaccinations including date and vaccine manufacturer, and demographic information were obtained via questionnaire (see **Table 1**, **Supplemental Tables S1, S2**). For the immunocompromised cohorts, 100% (30/30) of SARS-CoV-2 history positive participants had their infection confirmed by a positive laboratory PCR test. For the SARS-CoV-2 history positive immunocompetent individuals, 10/11 infections were confirmed by PCR and 1/11 was confirmed by a positive lateral flow test with accompanying symptoms.

**Table 1:** Participant demographic information.

	Rheumatology n = 29	Renal Transplant n = 46	PLWH n = 27	Immunocompetent n = 64
Sex				
Male	6	26	18	19
Female	23	20	9	45
Age (years)				
Mean (SD)	63 (13.2)	59 (13.8)	53 (11.2)	46 (15.5)
Median	63	61	50	42
Range (min, max)	22, 85	25, 80	36, 79	25, 75
Missing Data	0	0	0	11
Ethnicity				
White	27	44	19	61
Asian	2	0	2	3
Black	0	2	4	0
Other (including mixed)	0	0	2	0
Vaccine Type				
1st and 2nd dose				
Oxford/AstraZeneca	12	36	17	22
Pfizer/BioNTech	17	10	10	40
Moderna	0	0	0	0
Missing Data	0	0	0	2
3rd dose				
Oxford/AstraZeneca	0	2	0	0
Pfizer/BioNTech	25	41	22	38
Moderna	4	3	5	24
Missing Data	0	0	0	2
**4th dose**			N/A	N/A
Oxford/AstraZeneca	0	0		
Pfizer/BioNTech	8	24		
Moderna	1	2		
Janssen	0	0		
Missing Data	0	0		
Prior COVID				
Yes	6	16	8	12
No	23	30	19	52

### Peptides

The peptide pool consisted of 470 15mer peptides from the ancestral strain of SARS-CoV-2, overlapping by 11-amino acids, covering the entire proteome of the nucleocapsid phosphoprotein (Miltenyi Biotec, Bergisch Gladbach, Germany), membrane glycoprotein (Miltenyi Biotec) and the spike (S1 and S2) protein (JPT Peptide Technologies, Berlin, Germany). All peptides were purified by High Performance Liquid Chromatography. Previous studies demonstrated a concentration of 0.5µg/ml/peptide was sufficient to generate specific T cell responses (Oliver et al., 2022).

### Stimulation

Whole blood stimulations were carried out as previously described (Oliver et al., 2022). Briefly, a single 10ml sodium heparin vacutainer (Becton Dickinson, Franklin Lakes, New Jersey, USA) tube of blood was collected from each participant and 1ml whole blood samples were aliquoted into sterile T332 Micrewtubes (Simport Scientific, Saint-Mathieu-de-Beloeil, Canada) containing pre-aliquoted peptides, phytohaemagglutinin-L (positive control) or phosphate-buffered saline (PBS) (negative control). Samples were incubated at 37°C, 5% CO2, for 18-22 hours. Tubes were then centrifuged at 2000g for 3 minutes before harvesting ~200-300µl plasma from each blood sample. Plasma samples were stored at -20°C for up to 6 weeks prior to analysis.

### ELISA for interferon-gamma

IFN-γ protein was measured by an IFN-γ ELISA MAX Deluxe kit (BioLegend, San Diego, California, USA), following manufacturer’s instructions with a few modifications: an additional point on the standard curve (1000pg/ml); a 1-hour incubation for standards, samples, and blanks; and a pre-read step (at 450nm with just the TMB substrate) to standardise development of the assay. When standard 2 reached an optical density of 0.1, stop solution was added and the plate was read at 450nm. The amount of IFN-γ in each sample was analysed using the Gen5 software 8-point standard curve. To calculate the T cell response to SARS-CoV-2, the amount of IFN-γ in the control (PBS only) sample was subtracted from the corresponding value for the SARS-CoV-2 peptide stimulated sample and reported as pg/ml of plasma. In the absence of a response to the peptides, the amount of IFN-γ was calculated as below the lower limit of detection (LLOD). Therefore, a value equal to the lowest value on the standard curve was given for that sample.

### Luminex assay for interleukin-2

Cytokine IL-2 was measured by Bio-plex Pro Human Cytokine IL-2 Assays (Bio-Rad, Hercules, CA, USA). The median fluorescent intensity of the IL-2 bead set was measured on a Bio-plex 200 instrument (Bio-Rad). Cytokine concentration was calculated from a control curve of standard provided separately by the same supplier. In the absence of a response to the peptides, the amount of cytokine was considered as below the LLOD and a value equal to the lowest value on the standard curve was given for that sample.

### Chimeric ELISA for SARS-CoV-2 spike specific IgG

SARS-CoV-2 spike specific IgG was measured by an in-house assay adapted from the assay described in (Schuurman et al., 1997). Microtiter plates were coated with SARS-CoV-2 full-length spike protein (InBio, Charlottesville, VA, USA) at 0.5µg/well and incubated with plasma from unstimulated blood samples from all cohorts. Bound spike-specific IgG antibodies were detected using peroxidase conjugated Mouse anti-human IgG FCγ (Jackson ImmunoResearch, West Grove, PA, USA) at 0.08µg/well. Direct binding of IgG antibodies bound to the spike protein was quantified from a standard curve created using a mouse anti-Der p 2 chimeric human IgG1 antibody bound to nDer p 2 coated wells. Plasma samples obtained before the pandemic were used as negative controls.

### SARS-CoV-2 neutralising antibody assay

Live SARS-CoV-2 neutralisation assays were carried out as described previously (Fielding et al., 2022). Briefly, 600 plaque forming units of wild-type SARS-CoV-2 was incubated with 3-fold serial dilutions of plasma in duplicate for 1 hour at 37°C. Mixes were added to VeroE6 cells in 96-well plates, seeded 18 hours prior at 2x10^4^, for 48 hours at 37°C. Monolayers were fixed with 4% paraformaldehyde, permeabilised with 0.5% NP-40, then incubated in 3% blocking buffer (PBS containing 0.1% Tween and 3% non-fat milk) for 1 hour in the dark. Primary antibody (anti-nucleocapsid 1C7, Stratech, Ely, Cambridgeshire, UK) was added in 1% blocking buffer (PBS containing 0.1% Tween and 1% non-fat milk) for 1 hour at room temperature. Monolayers were washed with PBST (PBS containing 0.1% Tween), and secondary antibody (anti-mouse IgG-HRP, Pierce, Waltham, Massachusetts, USA) was added in 1% blocking buffer for 1 hour. Monolayers were washed with PBST, developed using OPD (OPD Tablets and Peroxidase substrate buffer; Fisher, Waltham, Massachusetts, USA), and read on a Clariostar Omega plate reader at 495nm. Control wells of no virus, virus but no antibody, and a standardised serum displaying moderate activity were included in each experiment. The 50% neutralising titre (NT50) values were calculated in GraphPad Prism 9 *v 9.5.0* using a 4-parameter nonlinear regression. Where no neutralisation was observed, an arbitrary value of 1.0 was given.

### Statistics

GraphPad Prism Version 9.5.0 was used for all statistical analyses of datasets.

Significance was determined using either a Kruskal-Wallis test with Dunn’s multiple comparison test or a Mann Whitney test. A p-value <0.05 was considered significant. Simple linear regression was used to determine Pearson R squared values and associated p values. For box and whisker plots: the middle line = median; lines of the box = 25^th^ and 75^th^ percentiles; whiskers = minimum and maximum values.

## Results

SARS-CoV-2 immune responses were analysed from participants who fell into one of 4 cohorts: rheumatology patients, renal transplant recipients, PLWH or immunocompetent participants. Participant demographics are summarised in **Table 1**. Rheumatology patients were established on biologic therapy (**Supplemental Table S1**), renal transplant patients were taking at least two immunosuppressants (**Supplemental Table S2**), and PLWH were all on HAART, had no AIDS diagnosis or were taking immunosuppressants at the time of blood donation.

### IFN-γ and IL-2 responses in SARS-CoV-2 infected and uninfected individuals following booster vaccinations

Blood samples from all participants were stimulated overnight with SARS-CoV-2 peptides and the level of IFN-γ and IL-2 was measured in the plasma by ELISA and a Luminex assay respectively (see methods).

**Figure 1:**
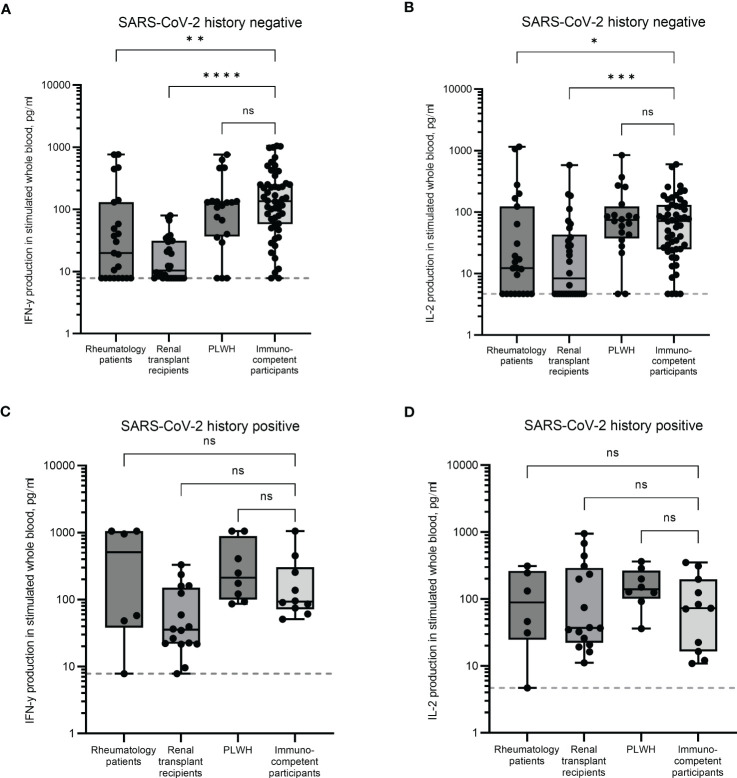
SARS-CoV-2-specific T cell responses in immunocompromised and immunocompetent cohorts. IFN-γ and IL-2 production in whole blood in response to overnight stimulation with SARS-CoV-2 peptide mega pool in individuals with no prior SARS-CoV-2 infection **(A, B)** and in individuals with a previous history of SARS-CoV-2 infection **(C, D)**. IFN-γ was measured by ELISA and IL-2 was measured by Luminex assay. Significant differences were calculated by Kruskal-Wallis test with Dunn’s multiple comparison test. *p<0.05, **p<0.01, ***p<0.001, ****p<0.0001, ns = not significant. Dotted line indicates lower limit of detection.

In individuals with no history of infection, significant differences were observed in the magnitude of IFN-γ responses. Rheumatology patients and renal transplant recipients had significantly lower median IFN-γ levels (19.8pg/ml and 10.3pg/ml, p < 0.01 and p < 0.0001, respectively) compared to immunocompetent participants (median IFN-γ of 135pg/ml, **Figure 1A**). Significant reductions in IL-2 were observed in both these groups (p < 0.05 and p < 0.001 for rheumatology and renal transplant cohorts, respectively). Median levels of IL-2 were 12.3pg/ml for the rheumatology cohort and 8.3pg/ml for the renal transplant cohort compared to immunocompetent controls (median IL-2 71.6pg/ml, **Figure 1B**). No differences in either IFN-γ or IL-2 production in the PLWH cohort were observed, with median levels for both cytokines being comparable to immunocompetent participants (**Figures 1A, B**).

Other variables that could have influenced T cell responses were considered. The type of initial vaccines that the participants received, and the gender of the participants did not significantly influence the T cell responses observed in any of the cohorts. However, there was ~2-4-fold lower median cytokine response in the rheumatology and renal transplant cohorts in those receiving an adenovirus initial vaccine than those receiving an mRNA initial vaccine (**Supplemental Figures S1A, S1B**, **Supplemental Table S4**). The age of the immunocompromised participants did not influence the T cell responses. A slight reduction in IFN-γ production was observed in the immunocompetent cohort as the participants got older (R^2 ^= 0.08239, p = 0.0455, **Supplemental Table S3**).

These results indicate that uninfected individuals being treated for rheumatology conditions and those having received a kidney transplant have an impaired T cell immune response following multiple vaccinations compared to PLWH and immunocompetent controls.

In previously infected individuals, cytokine production was comparable between all groups (**Figures 1C, D**). The median IFN-γ levels in the renal transplant cohort were ~2.6 fold lower than the immunocompetent group (35.4pg/ml and 92.8pg/ml respectively), although this was not statistically significant.

### Antibody responses in SARS-CoV-2 infected and uninfected individuals following booster vaccinations

**Figure 2:**
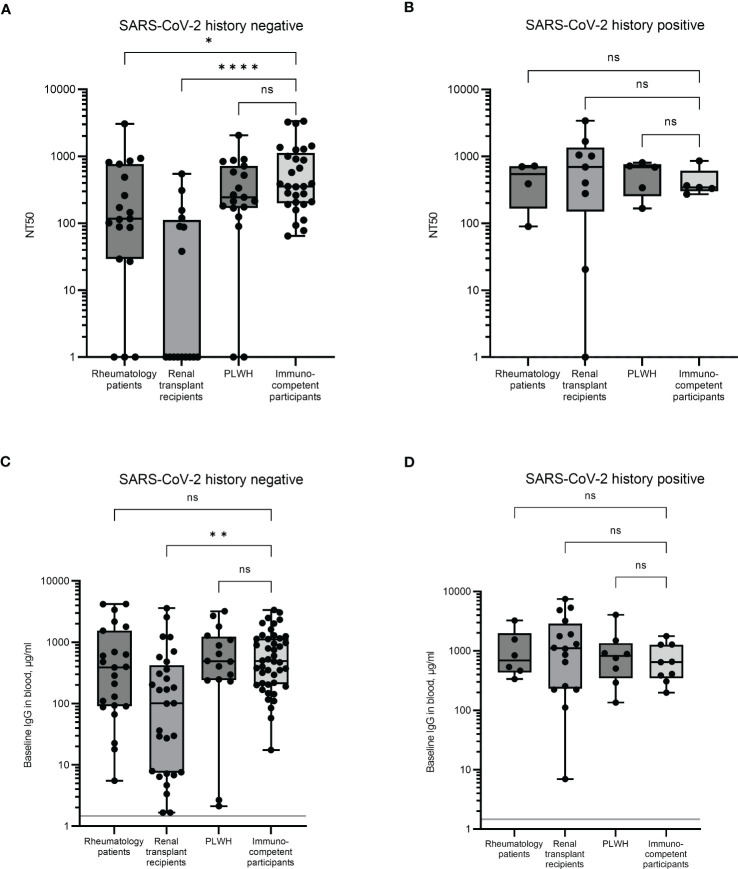
Serological immune responses in immunocompromised and immunocompetent cohorts. Neutralising antibody levels were determined by NT50 in uninfected **(A)** and previously infected **(B)** individuals. When no neutralisation was observed, an arbitrary value of 1 was assigned. Baseline levels of SARS-CoV-2 full length spike-specific IgG antibodies were measured in uninfected **(C)** and previously infected **(D)** individuals. Significant differences were calculated by Kruskal-Wallis test with Dunn’s multiple comparison test. *p<0.05, **p<0.01, ****p<0.0001, ns = not significant. Solid grey line indicates the mean IgG concentration of three pre-pandemic plasma samples.

Unstimulated plasmas from the same blood samples above were analysed for neutralising antibody levels using the NT50. Due to the difficulties implementing the neutralising antibody assay in large quantities, a subset of samples was analysed for each cohort. To limit bias and cover as wide a range of immune responses as possible, the samples were selected based on low, medium, and high IFN-γ and IL-2 responses. In individuals with no history of infection, a significant difference was observed in the magnitude of the neutralising antibody responses in renal transplant recipients compared to immunocompetent controls (p < 0.0001, **Figure 2A**). The renal transplant group had a median NT50 of 1.0 (an arbitrary value assigned when no neutralisation was observed) with over 50% of the participants unable to mount a neutralising antibody response, in comparison to the immunocompetent participants who had a median NT50 of 355. A significant reduction was also observed in the rheumatology patient cohort (median NT50 = 116, p < 0.05), although in this group, only 16% of donors did not mount a detectable neutralising antibody response (**Figure 2A**). No differences in neutralising antibody responses were observed in the PLWH cohort. In previously infected individuals, neutralising antibody levels were comparable between all groups (**Figure 2B**).

Plasmas from all participants were analysed for SARS-CoV-2 full-length spike specific IgG and correlated with the NT50 result. Significant correlation between results was observed in all four groups (**Supplemental Figure S2**), suggesting that the spike specific IgG antibody level is a good proxy for functional antibody responses. However, despite this correlation, there was no significant difference observed in IgG levels between the rheumatology patients and the immunocompetent participants (**Figure 2C**). Furthermore, although the responses in the renal transplant recipient group were still significantly reduced compared to the immunocompetent cohort (p = 0.01, mean IgG levels were 101.2µg/ml and 492.4µg/ml, respectively), the magnitude of this difference was much larger when measuring neutralisation as opposed to IgG levels. Thus, both the quality and the quantity of IgG may be affected in these individuals. As with the NT50 response, no significant difference was observed between the PLWH and the immunocompetent cohort.

These results suggest that renal transplant recipients, and to a lesser extent, rheumatology patients, have an impaired antibody response following multiple vaccinations compared to PLWH and immunocompetent controls. In previously infected individuals, IgG antibody levels were comparable between all groups (**Figure 2D**).

Other variables that could have influenced antibody responses were also assessed. The type of initial vaccines received, age, and gender of the participants did not influence the antibody responses observed in the renal transplant or the PLWH cohorts (**Supplemental Figure S1C**, **Supplemental Tables S3, S4**). In the rheumatology cohort, participants who received mRNA vaccines as their initial doses had a 2.2-fold higher median IgG response than those receiving adenovirus vaccines initially. Contrastingly, immunocompetent participants who initially received adenovirus vaccines had a 2.7-fold higher IgG response than those who received mRNA vaccines initially (**Supplemental Figure S2C**). Females in the rheumatology group also showed a 5-fold higher response than males in the same group, although the ratio of males to females in this group was highly skewed toward females which may have influenced this observation (**Table 1**, **Supplemental Table S4**).

### Immune responses in rheumatology patients receiving different biologic drugs

**Figure 3:**
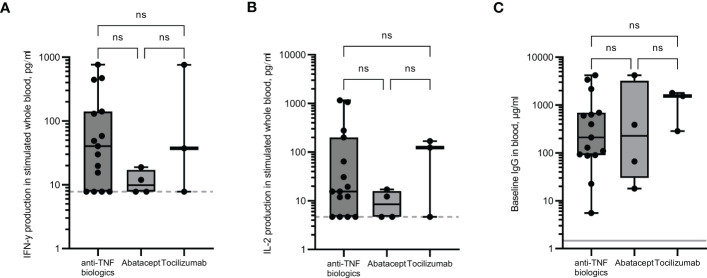
Immune responses in rheumatology patients receiving different anti-rheumatic drugs. IFN-γ **(A)** and IL-2 **(B)** production in whole blood in response to overnight peptide stimulation and baseline measurement of spike specific IgG antibodies **(C)** in individuals receiving different anti-rheumatic drugs. IFN-γ and IgG were measured by ELISA and IL-2 was measured by Luminex assay. Statistical analysis was performed with a Kruskal-Wallis test with Dunn’s multiple comparison test. ns = not significant. Dotted line indicates lower limit of detection. Solid grey line indicates the mean IgG concentration of three pre-pandemic plasma samples.

Responses in the rheumatology cohort were assessed depending on the biologic drugs they received. Individuals on abatacept (a selective co-stimulation immunomodulator) showed reduced levels of IFN-γ and IL-2 production following whole blood stimulation (**Figures 3A, B**) compared to those on αTNF biologics or tocilizumab (TCZ, an αIL-6R monoclonal antibody), although numbers were too small to be statistically significant. A positive T cell response was seen in only 50% of patients on abatacept, compared to 85% and 67% for those on anti-TNF biologics and TCZ, respectively. No significant differences in baseline IgG levels were observed (**Figure 3C**).

### Immune responses in renal transplant recipients receiving different combinations of immunosuppressive drugs

**Figure 4:**
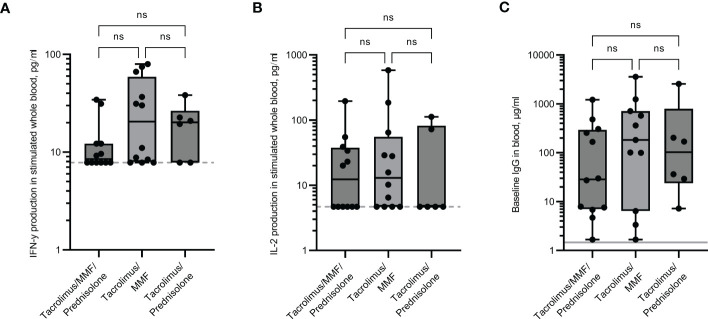
Immune responses in renal transplant recipients receiving different combinations of immunosuppressive drugs. IFN-γ **(A)** and IL-2 **(B)** production in whole blood in response to overnight peptide stimulation and baseline measurement of spike specific IgG antibodies **(C)** in individuals receiving different combinations of immunosuppressive drugs. MMF = Mycophenolate mofetil. IFN-γ and IgG were measured by ELISA and IL-2 was measured by Luminex assay. Statistical analysis was performed with a Kruskal-Wallis test with Dunn’s multiple comparison test. ns = not significant. Dotted line indicates lower limit of detection. Solid grey line indicates the mean IgG concentration of three pre-pandemic plasma samples.

Responses in the renal transplant cohort were assessed depending on the type of immunosuppressive drugs they received. Individuals who received three immunosuppressive drugs (Tacrolimus, Mycophenolate mofetil (MMF) and Prednisolone) had a lower IFN-γ response and IgG baseline levels than those on two drugs only, although numbers were too small to be significant (**Figures 4A, C**). The median IFN-γ responses for those receiving three drugs were 2.2-fold lower than those receiving just Tacrolimus and MMF, or Tacrolimus and Prednisolone. Median IgG levels were 29.8µg/ml for those receiving three drugs and 182.5µg/ml and 102.7µg/ml for those receiving just Tacrolimus and MMF, or Tacrolimus and Prednisolone respectively. The median IL-2 levels were comparably low in all three groups (**Figure 4B**).

### Immune responses in the PWLH cohort relative to CD4 cell count

**Figure 5:**
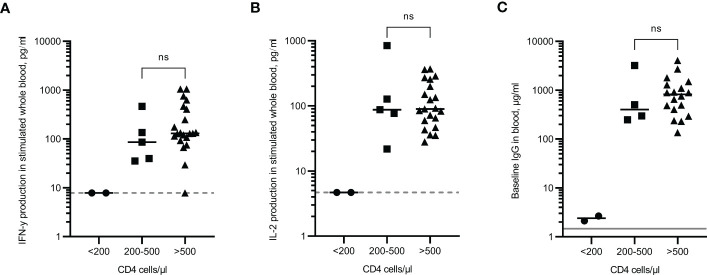
Immune responses in PLWH according to CD4 cell count. IFN-γ **(A)** and IL-2 **(B)** production in whole blood in response to overnight peptide stimulation and baseline measurement of spike specific IgG antibodies **(C)** in PLWH. ● = CD4 cell count of <200 cells/µl, ⏹ = CD4 cell count between 200 and 500 cells/µl, ▲ = CD4 cell count of >500 cells/µl. Results show individual results with median. IFN-γ and IgG were measured by ELISA and IL-2 was measured by Luminex assay. Statistical analysis was performed with a Mann Whitney test. ns = not significant. Dotted line indicates lower limit of detection. Solid grey line indicates the mean IgG concentration of three pre-pandemic plasma samples.

The results from participants in the PLWH cohort were analysed according to their most recent CD4 count; those with a CD4 count less than 200 cells/µl (increased risk of opportunistic infections), those with a CD4 count between 200-500 cells/µl (lower than the ‘normal’ range of uninfected individuals but opportunistic infections are unlikely), and those above 500 cells/µl (considered a ‘normal’ range). The two individuals with CD4 counts <200 cells/µl did not demonstrate any T cell response (both IFN-γ and IL-2 were <LLOD) and had extremely low IgG levels (**Figures 5A–C**). These individuals also had an NT50 result of 1, an arbitrary value that was assigned when no antibody neutralisation of the virus was observed (**Figure 2A**). These results indicate that no immune response had been generated following multiple vaccinations. One individual with a CD4 count >500 cells/µl was reported as having a <LLOD result for IFN-γ (**Figure 5A**). Despite this individual having a positive response to the viral peptides, there was also a high background level of IFN-γ in the negative control which when subtracted, led to the result being below the assay limit of detection. However, given that IFN-γ was produced in response to the peptides, along with a positive IL-2 result and an IgG level of 238.3µg/ml, it would be predicted that this person does not have an impaired immune response following vaccinations. No significant differences were observed in the magnitude of cytokine production or IgG levels between individuals with CD4 cell counts between 200-500 cells/µl and individuals of CD4 cell counts >500 cells/µl. Statistical analysis was not performed on the group containing <200 cells/μl due to only two participants in that category.

### Immune response comparison between 3^rd^ and 4^th^ vaccination doses in immunocompromised cohorts

**Figure 6:**
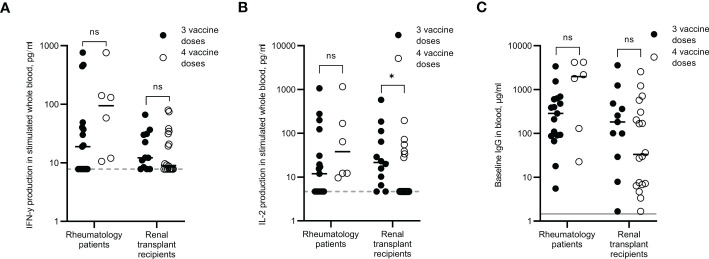
Immune responses in rheumatology patients and renal transplant recipients after three or four SARS-CoV-2 vaccine doses. IFN-γ **(A)** and IL-2 **(B)** production in whole blood in response to overnight peptide stimulation and baseline measurement of spike-specific IgG antibodies **(C)** in individuals who received three SARS-COV-2 vaccine doses (●) or individuals who received four SARS-CoV-2 vaccine doses (O). Results show individual results with median. IFN-γ and IgG were measured by ELISA and IL-2 was measured by Luminex assay. Statistical analysis was performed with a Mann Whitney test. *p<0.05, ns = not significant. Dotted line indicates lower limit of detection. Solid grey line indicates the mean IgG concentration of three pre-pandemic plasma samples.

Participants from the rheumatology and renal transplant cohorts were split into those that had received a third vaccination dose and those that had received a fourth vaccination dose. Although the samples were not all taken at the same time point since vaccination, regression analysis revealed no correlation between number of days since vaccination and levels of IFN-γ, IL-2 or IgG (R-squared values = 0.227, 0.017 and 0.066 respectively for rheumatology patients, and 0.009, 0.001 and 0.055 respectively for renal transplant recipients, data not shown). In the rheumatology cohort, responses were increased in those that had received a fourth dose compared to third dose only. Median IFN-γ, IL-2 and IgG levels were 5-fold, 3.2-fold and 6.9-fold higher respectively following a fourth dose, although none of these increases were statistically significant, most likely due to the small number for the fourth dose group (**Figures 6A–C**). Detectable IFN-γ and IL-2 levels were observed for 100% of rheumatology patients who received a fourth dose, compared to 53% and 41% respectively for those who received just three doses.

In the renal transplant cohort, the cytokine responses remained low even after receiving a fourth dose, with only 55% of participants recording a positive IFN-γ response and only 33% of them recording a positive IL-2 response. IgG levels did not appear to be boosted following a fourth dose either, with just 17% of participants recording an IgG level >10µg/ml.

## Discussion

Whilst pre-pandemic levels of social normality have returned for many people, immunocompromised individuals may still feel particularly vulnerable to SARS-CoV-2 related complications and potential associated social isolation. Given the conflicting or unavailable information regarding responses to vaccinations and the level of protection, or lack of protection that these provide, immunocompromised individuals need more information on whether these feelings are justified. In this study, we have simultaneously taken three immunocompromised cohorts and assessed their cellular and humoral immune responses to multiple SARS-CoV-2 vaccination doses from one blood sample, revealing impaired T cell and humoral immune responses in rheumatology patients and renal transplant recipients compared to PLWH and immunocompetent individuals.

### Humoral responses

When assessing the antibody response, NT50 against live virus was used as a functional assay for neutralising antibodies, and total IgG was used for circulating antibody levels. Here we demonstrated strong correlations between the two readouts, a finding that has been observed by others (Amanat et al., 2020; Suthar et al., 2020). However, despite the good correlation, differences between the assays were revealed. In the uninfected group of rheumatology patients, three individuals were identified as having no detectable levels of neutralising antibodies. Overall, the median NT50 level was significantly lower than the control group. When comparing total IgG, this difference was no longer significant, although two out of the three individuals with no neutralising antibodies also had the lowest IgG levels. Amongst transplant recipients, circulating anti-spike IgG was detected in individuals who did not mount a neutralising response. It is plausible that affinity maturation is partially inhibited in these cohorts, leading to production of lower affinity antibodies and therefore weaker neutralisation. However, providing a definitive explanation for the observed results is beyond the scope of this study. The NT50 result is an important functional marker and a more sensitive one, yet total IgG readings are a good substitute when the NT50 assay is not feasible. Interestingly, antibody levels in individuals from both cohorts who had a previous SARS-CoV-2 infection were comparable with previously infected immunocompetent individuals, indicating that a humoral immune response can be mounted in response to natural infection, but it is impaired in response to vaccinations. This may indicate that the design of the vaccine doses administered in this study could be improved as they do not mimic natural infection in certain individuals.

### T cell responses

Whole blood peptide-stimulation assays have become a widely utilised method for investigating SARS-CoV-2 specific T cell responses due to the relative simplicity, speed and small amount of blood required (Murugesan et al., 2020; Lineburg et al., 2021; Petrone et al., 2021; Tan et al., 2021; Oliver et al., 2022). Our data revealed that after three or four vaccination doses, T cell responses were significantly lower in patients with rheumatological conditions than those of immunocompetent controls in uninfected individuals. One infected individual did not record a positive IFN-γ, and another infected individual did not record a positive IL-2 response. This highlights the importance of measuring both cytokines to assess T cell responses, particularly in immunocompromised groups where immune responses may be atypical. Results presented here for renal transplant recipients revealed similar findings, with uninfected individuals displaying markedly reduced T cell activation in response to SARS-CoV-2 peptide stimulation. As with the antibody responses, individuals from the immunocompromised cohorts who had been naturally infected did not have an impaired T cell response compared to immunocompetent controls, further emphasising discord between naturally induced immune responses and vaccine induced immune responses. This implies that even with a weakened immune system, a memory response to SARS-CoV-2 can be produced by individuals within these immunocompromised cohorts, indicating that a different type of vaccine such as the live attenuated vaccines that are currently being developed may be beneficial to these groups (Trimpert et al., 2021; Chen et al., 2022; Liu et al., 2022; Tang et al., 2022).

Of interest, two previously infected patients in the renal transplant cohort had no/low IFN-γ responses, but the IL-2 levels in these patients were positive, further strengthening the argument for measuring two different cytokines to assess T cell responses. These two patients with low IFN-γ were not the same patients with no/low NT50 results. This is an important finding given that regression analysis in this group revealed excellent correlation between IFN-γ and IgG levels (p < 0.0001, data not shown), and highlights the potential utility for measuring serological responses and T cell responses concurrently to provide a more accurate picture of an individual’s immune status.

### Rheumatology patients

The types of drugs used to treat inflammatory conditions may impair immune responses to vaccines such as influenza, herpes zoster, hepatitis B and others (Friedman et al., 2021). The effects of different disease-modifying anti-rheumatic drugs on SARS-CoV-2 vaccines are still being examined, but evidence so far suggests that rituximab and abatacept have significant adverse effects on immune response; methotrexate has lesser negative effect; whereas anti-TNF biologics and other biologics such as tocilizumab have minimal adverse effects (Friedman et al., 2021; Saleem et al., 2022; Simon et al., 2022). Although the numbers in our rheumatology group are too small for analysis to be of significance, our data agrees with findings that for T cell responses, patients receiving abatacept have the lowest median levels of both IFN-γ and IL-2. Small studies have demonstrated that when methotrexate was temporarily withdrawn for 1-2 weeks after vaccine administration, vaccine immunogenicity was improved (Araujo et al., 2022; Azzolini et al., 2022). Temporarily suspending treatment can result in disease flare ups, and although often mild, patients may express hesitancy over this intervention (Xie et al., 2022). Therefore, each patient will need to weigh the risks versus benefits of pausing treatment temporarily to potentially increase vaccine immunogenicity and reduce the likelihood of requiring SARS-CoV-2 related hospitalisation (Agrawal et al., 2022; Lee et al., 2023).

### Renal transplant recipients

There is currently limited data on vaccine immunogenicity in SOTR following four or even five doses of COVID-19 vaccines. A fourth dose has been associated with a slight improvement in humoral responses in individuals with no or a low response after three doses (Osmanodja et al., 2022; Peghin et al., 2022). One study found that serological immune responses only increased from 55.6% after four doses to 57.5% following a fifth vaccine dose (Osmanodja et al., 2022). Results from our renal transplant recipients did not demonstrate that a fourth dose elicited improved responses above those individuals who had three doses, with only 55% of participants recording a positive IFN-γ response, 33% recording a positive IL-2 response and just 17% of participants recording an IgG level >10µg/ml. Further follow-ups of these non-responders after subsequent doses will be an important consideration to determine if they will elicit an immune response or if they are chronically unresponsive. If the latter, a different protective approach may be required.

Predictors of poor antibody responses in renal transplant patients have been previously reported for those receiving triple immunosuppression and/or those receiving MMF (Stumpf et al., 2021; Smith et al., 2022). Our results demonstrated that those on three immunosuppressive drugs have a 2.2-fold lower IFN-γ response and 3.4 to 6.1-fold lower IgG levels than those receiving only two immunosuppressors. IL-2 responses were comparable, further strengthening the need to measure at least two cytokines when assessing the T cell SARS-CoV-2 specific immune response.

There has been conflicting data reported on immune responses in SOTR depending on vaccine type the recipients received, with some evidence suggesting Moderna is superior to Pfizer-BioNTech for seroconversion, and that Pfizer-BioNTech was superior to ChAdOx1 for the development of humoral responses but comparable for T cell responses (Prendecki et al., 2021; Stumpf et al., 2021; Smith et al., 2022). Our renal transplant cohort received either ChAdOx1 or Pfizer-BioNTech as their initial two doses, followed by various combinations of ChAdOx1, Pfizer-BioNTech or Moderna for their additional vaccine doses. No significant differences were seen for the development of either humoral or T cell responses depending on if ChAdOx1 or Pfizer-BioNTech was administered, and given that so many of this cohort did not develop vaccine-induced immune responses even after 4 doses, perhaps the more important question should be, would a different type of vaccine produce better results in these immunocompromised cohorts?

### PLWH

Results from the PLWH cohort revealed that following three vaccination doses, both T cell responses and antibody responses are comparable to immunocompetent controls in uninfected individuals. Responses were also comparable in previously infected individuals. In the uninfected group of PLWH, there were two individuals who did not record a detectable cytokine response or a neutralising antibody response. PLWH that have a CD4 count less than 200 cells/µl have an increased risk of opportunistic infections. These individuals had a lower percentage of vaccine-responding T cells than those with counts above 200 cells/µl (Tortellini et al., 2022). In agreement with this, the 2 individuals in our cohort with impaired vaccine-induced immune responses had CD4 counts of <200cells/µl, suggesting that assessment of total CD4 cell count should be considered when determining vaccine efficacy (Alrubayyi et al., 2021; Lv et al., 2022; Mullender et al., 2022). It is important to note that all the participants in this cohort had controlled viremia and none had high viral load counts (all under 500 copies/ml) so these results cannot be extrapolated to individuals with uncontrolled viremia or high viral loads.

### General discussion

Certain immunocompromised individuals remain at higher risk of breakthrough infections, severe disease and SARS-CoV-2 related hospitalisations despite receiving multiple vaccinations. The major limitations of our study include limited ethnic demographics, relatively low numbers for comparing anti-inflammatory or immunosuppressive drug effects, no participants in the PLWH cohort with uncontrolled viremia, no confirmation of natural infection status via an anti-N/anti-ORF8 ELISA lab test to rule out asymptomatic infections in the history negative groups, and different time lapses between the last vaccine dose and blood collection. Despite these limitations, the results support the growing argument that specific subgroups within each immunocompromised cohort could benefit from distinct, personalised immunisation strategies (Alrubayyi et al., 2021; Karaba et al., 2022; Mullender et al., 2022). For example, a pause or a switch in certain drug treatments may be necessary to elicit the most immunogenic response when booster vaccinations are administered (Stumpf et al., 2021; Araujo et al., 2022; Azzolini et al., 2022; Osmanodja et al., 2022; Saleem et al., 2022). These assays may be a relatively simple way of identifying those individuals who do not respond to vaccines and thus are more at risk of adverse outcomes from SARS-CoV-2 infection. These individuals may benefit from enhanced isolation during periods of COVID surges or high transmission or could be ideal candidates for receiving primary neutralising monoclonal antibodies prophylactically and/or prioritised for early antiviral or monoclonal antibody therapy if they get infected.

Levels of circulating SARS-CoV-2 specific IgG antibodies do not always correlate significantly with IL-2 or IFN-γ production (data not shown and (Alrubayyi et al., 2021)). Furthermore, neutralising antibodies to the original Wuhan strain are functionally reduced against other viral strains, including Omicron (Zhang et al., 2021; Dejnirattisai et al., 2022; Lyke et al., 2022). Whilst using the full-length spike protein from the original Wuhan strain in these IgG ELISA assays is informative, there is a possibility that antibody responses to other variants may be even further diminished in the immunocompromised groups studied here. In contrast, T cell responses are generally longer lived than antibody responses (Guo et al., 2022; Moore et al., 2023) and T cell memory encompasses a broad recognition of viral proteins, thus T cell responses remain robust against viral variants, including Omicron (Moss, 2022; Oliver et al., 2022). Consequently, the argument for prioritising the assessment of T cell responses in immunocompromised cohorts to provide the best preventative strategy can be made.

Finally, given that there is no consensus on what level of cellular or humoral responses actually constitutes protective immunity from severe or moderate disease, or how long this protection lasts in the general population, it is equally difficult to define protective immunity in immunocompromised individuals (Prendecki et al., 2021; Mullender et al., 2022; Parker et al., 2022).

## Conclusions

In this study, we revealed that in comparison to immunocompetent individuals, rheumatology patients and renal transplant recipients had impaired T cell and humoral immune responses even after multiple SARS-CoV-2 vaccinations. Conversely, PLWH had comparable responses. We provide evidence that measuring serologic and cellular aspects of SARS-CoV-2 immunity builds a complete picture of vaccine efficacy and immunogenicity in different immunocompromised cohorts. Assessment of immune responses to identify vaccine-non responders as demonstrated here, could be critical to protecting those most at risk by providing the opportunity to offer alternative preventive strategies instead of repeated vaccinations to which they might never respond.

## Data availability statement

The raw data supporting the conclusions of this article will be made available by the authors, without undue reservation.

## Ethics statement

The studies involving human participants were reviewed and approved by Wales Research Ethics Committee (REC) 6. The patients/participants provided their written informed consent to participate in this study.

## Author contributions

MO, HM, and DC conceptualised the study. MO, DC, HM, RY, RS and SA designed the study and associated experiments. MO, DC, AA-O and RY gained ethical approval for the study. DC, MS, NH, AA-O, RY, RM and MO recruited participants. RM, MB, KB and MO acquired and analysed the data. MO wrote the manuscript. All authors reviewed, commented on, and edited the manuscript.

## References

[B1] Abbasi J. (2021). Researchers tie severe immunosuppression to chronic covid-19 and virus variants. Jama 325, 2033–2035. doi: 10.1001/jama.2021.7212 33950236

[B2] Agrawal U. Bedston S. Mccowan C. Oke J. Patterson L. Robertson C.. (2022). Severe covid-19 outcomes after full vaccination of primary schedule and initial boosters: pooled analysis of national prospective cohort studies of 30 million individuals in England, northern Ireland, Scotland, and Wales. Lancet 400, 1305–1320. doi: 10.1016/S0140-6736(22)01656-7 36244382 PMC9560746

[B3] Alrubayyi A. Gea-Mallorqui E. Touizer E. Hameiri-Bowen D. Kopycinski J. Charlton B.. (2021). Characterization of humoral and sars-Cov-2 specific T cell responses in people living with hiv. Nat. Commun. 12, 5839. doi: 10.1038/s41467-021-26137-7 34611163 PMC8492866

[B4] Amanat F. Stadlbauer D. Strohmeier S. Nguyen T. H. O. Chromikova V. Mcmahon M.. (2020). A serological assay to detect sars-Cov-2 seroconversion in humans. Nat. Med. 26, 1033–1036. doi: 10.1038/s41591-020-0913-5 32398876 PMC8183627

[B5] Araujo C. S. R. Medeiros-Ribeiro A. C. Saad C. G. S. Bonfiglioli K. R. Domiciano D. S. Shimabuco A. Y.. (2022). Two-week methotrexate discontinuation in patients with rheumatoid arthritis vaccinated with inactivated sars-Cov-2 vaccine: a randomised clinical trial. Ann. Rheum Dis. 81, 889–897. doi: 10.1136/annrheumdis-2021-221916 35193873

[B6] Azzolini E. Pozzi C. Germagnoli L. Oresta B. Carriglio N. Calleri M.. (2022). Mrna covid-19 vaccine booster fosters b- and T-cell responses in immunocompromised patients. Life Sci. Alliance 5, e202201381. doi: 10.26508/lsa.202201381 35169017 PMC8860093

[B7] Belsky J. A. Tullius B. P. Lamb M. G. Sayegh R. Stanek J. R. Auletta J. J. (2021). Covid-19 in immunocompromised patients: a systematic review of cancer, hematopoietic cell and solid organ transplant patients. J. Infect. 82, 329–338. doi: 10.1016/j.jinf.2021.01.022 33549624 PMC7859698

[B8] Chen J. Wang P. Yuan L. Zhang L. Zhang L. Zhao H.. (2022). A live attenuated virus-based intranasal covid-19 vaccine provides rapid, prolonged, and broad protection against sars-Cov-2. Sci. Bull. (Beijing) 67, 1372–1387. doi: 10.1016/j.scib.2022.05.018 35637645 PMC9134758

[B9] Dejnirattisai W. Huo J. Zhou D. Zahradnik J. Supasa P. Liu C.. (2022). Sars-Cov-2 omicron-B.1.1.529 leads to widespread escape from neutralizing antibody responses. Cell 185, 467–484.E15. doi: 10.1016/j.cell.2021.12.046 35081335 PMC8723827

[B10] Farroni C. Aiello A. Picchianti-Diamanti A. Lagana B. Petruccioli E. Agrati C.. (2022). Booster dose of sars-Cov-2 messenger rna vaccines strengthens the specific immune response of patients with rheumatoid arthritis: a prospective multicenter longitudinal study. Int. J. Infect. Dis. 125, 195–208. doi: 10.1016/j.ijid.2022.10.035 36328289 PMC9622025

[B11] Ferreira V. H. Solera J. T. Hu Q. Hall V. G. Arbol B. G. Rod Hardy W.. (2022). Homotypic and heterotypic immune responses to omicron variant in immunocompromised patients in diverse clinical settings. Nat. Commun. 13, 4489. doi: 10.1038/s41467-022-32235-x 35927279 PMC9352686

[B12] Fielding C. A. Sabberwal P. Williamson J. C. Greenwood E. J. D. Crozier T. W. M. Zelek W.. (2022). Sars-Cov-2 host-shutoff impacts innate nk cell functions, but antibody-dependent nk activity is strongly activated through non-spike antibodies. Elife 11, e74489. doi: 10.7554/eLife.74489.sa2 35587364 PMC9239683

[B13] Frater J. Ewer K. J. Ogbe A. Pace M. Adele S. Adland E.. (2021). Safety and immunogenicity of the Chadox1 ncov-19 (Azd1222) vaccine against sars-Cov-2 in hiv infection: a single-arm substudy of a phase 2/3 clinical trial. Lancet HIV 8, E474–E485. doi: 10.1016/S2352-3018(21)00103-X 34153264 PMC8213361

[B14] Freer J. Mudaly V. (2022). Hiv and covid-19 in south Africa. Bmj 376, e069807. doi: 10.1136/bmj-2021-069807 35086921

[B15] Friedman M. A. Curtis J. R. Winthrop K. L. (2021). Impact of disease-modifying antirheumatic drugs on vaccine immunogenicity in patients with inflammatory rheumatic and musculoskeletal diseases. Ann. Rheum Dis. 80, 1255–1265. doi: 10.1136/annrheumdis-2021-221244 34493491 PMC8494475

[B16] Guo L. Wang G. Wang Y. Zhang Q. Ren L. Gu X.. (2022). Sars-Cov-2-Specific antibody and T-cell responses 1 year after infection in people recovered from covid-19: a longitudinal cohort study. Lancet Microbe 3, e348–e356. doi: 10.1016/S2666-5247(22)00036-2 35345417 PMC8942480

[B17] Isnardi C. A. Cerda O. L. Landi M. Cruces L. Schneeberger E. E. Montoro C. C.. (2022). Immune response to sars-Cov-2 third vaccine in patients with rheumatoid arthritis who had no seroconversion after primary 2-dose regimen with inactivated or vector-based vaccines. J. Rheumatol 49, 1385–1389. doi: 10.3899/jrheum.220469 36182107

[B18] Jyssum I. Kared H. Tran T. T. Tveter A. T. Provan S. A. Sexton J.. (2022). Humoral and cellular immune responses to two and three doses of sars-Cov-2 vaccines in rituximab-treated patients with rheumatoid arthritis: a prospective, cohort study. Lancet Rheumatol 4, e177–e187. doi: 10.1016/S2665-9913(21)00394-5 34977602 PMC8700278

[B19] Karaba A. H. Zhu X. Liang T. Wang K. H. Rittenhouse A. G. Akinde O.. (2022). A third dose of sars-Cov-2 vaccine increases neutralizing antibodies against variants of concern in solid organ transplant recipients. Am. J. Transplant. 22, 1253–1260. doi: 10.1111/ajt.16933 34951746 PMC8983554

[B20] Kolb T. Fischer S. Muller L. Lubke N. Hillebrandt J. Andree M.. (2021). Impaired immune response to sars-Cov-2 vaccination in dialysis patients and in kidney transplant recipients. Kidney360 2, 1491–1498. doi: 10.34067/KID.0003512021 35373105 PMC8786134

[B22] Lee L. Y. W. Tilby M. Starkey T. Ionescu M. C. Burnett A. Hattersley R.. (2023). Association of sars-Cov-2 spike protein antibody vaccine response with infection severity in patients with cancer: a national covid cancer cross-sectional evaluation. JAMA Oncol. 9, 188–196. doi: 10.1001/jamaoncol.2022.5974 36547970 PMC9936347

[B21] Lee A. Wong S. Y. Chai L. Y. A. Lee S. C. Lee M. X. Muthiah M. D.. (2022). Efficacy of covid-19 vaccines in immunocompromised patients: systematic review and meta-analysis. Bmj 376, e068632. doi: 10.1136/bmj-2021-068632 35236664 PMC8889026

[B23] Lineburg K. E. Neller M. A. Ambalathingal G. R. Le Texier L. Raju J. Swaminathan S.. (2021). Rapid whole-blood assay to detect sars-Cov-2-Specific memory T-cell immunity following a single dose of astrazeneca Chadox1-s covid-19 vaccine. Clin. Transl. Immunol. 10, e1326. doi: 10.1002/cti2.1326 PMC8360255 34408875

[B24] Liu Y. Zhang X. Liu J. Xia H. Zou J. Muruato A. E.. (2022). A live-attenuated sars-Cov-2 vaccine candidate with accessory protein deletions. Nat. Commun. 13, 4337. doi: 10.1038/s41467-022-31930-z 35896528 PMC9326133

[B25] Lu L. Chan C. Y. Chan-Ng P. P. L. Than M. Tan P. S. Y. Lim L. K.. (2023). Heterogenous antibody and T-cell responses to sars-Cov-2 mrna vaccines among immunocompromised young people. Clin. Transl. Med. 13, e1183. doi: 10.1002/ctm2.1183 36658466 PMC9852384

[B26] Lv Z. Li Q. Feng Z. Zheng X. Nayin Yang H.. (2022). Inactivated sars-Cov-2 vaccines elicit immunogenicity and T-cell responses in people living with hiv. Int. Immunopharmacol 102, 108383. doi: 10.1016/j.intimp.2021.108383 34824035 PMC8599017

[B27] Lyke K. E. Atmar R. L. Islas C. D. Posavad C. M. Szydlo D. Paul Chourdhury R.. (2022). Rapid decline in vaccine-boosted neutralizing antibodies against sars-Cov-2 omicron variant. Cell Rep. Med. 3, 100679. doi: 10.1016/j.xcrm.2022.100679 35798000 PMC9212999

[B28] Marra A. R. Kobayashi T. Suzuki H. Alsuhaibani M. Tofaneto B. M. Bariani L. M.. (2022). Short-term effectiveness of covid-19 vaccines in immunocompromised patients: a systematic literature review and meta-analysis. J. Infect. 84, 297–310. doi: 10.1016/j.jinf.2021.12.035 34982962 PMC8720049

[B29] Mishra M. Zahra A. Chauhan L. V. Thakkar R. Ng J. Joshi S.. (2022). A short series of case reports of covid-19 in immunocompromised patients. Viruses 14, 934. doi: 10.3390/v14050934 35632677 PMC9145915

[B30] Moore S. C. Kronsteiner B. Longet S. Adele S. Deeks A. S. Liu C. (2023). Evolution of long-term vaccine-induced and hybrid immunity in healthcare workers after different covid-19 vaccine regimens. Med. (N Y) 4, 191–215. doi: 10.1016/j.medj.2023.02.004 PMC9933851 36863347

[B31] Moss P. (2022). The T cell immune response against sars-Cov-2. Nat. Immunol. 23, 186–193. doi: 10.1038/s41590-021-01122-w 35105982

[B32] Mullender C. Da Costa K. A. S. Alrubayyi A. Pett S. L. Peppa D. (2022). Sars-Cov-2 immunity and vaccine strategies in people with hiv. Oxford Open Immunol. 3, iqac005. doi: 10.1093/oxfimm/iqac005 PMC9452103 36846557

[B33] Murugesan K. Jagannathan P. Pham T. D. Pandey S. Bonilla H. F. Jacobson K.. (2020). Interferon-gamma release assay for accurate detection of sars-Cov-2 T cell response. Clin. Infect. Dis. 73, e3130–e3132. doi: 10.1093/cid/ciaa1537 PMC7665338 33035306

[B34] Nadesalingam A. Cantoni D. Aguinam E. T. Chan A. C. Paloniemi M. Ohlendorf L.. (2022). Vaccination and protective immunity to sars-Cov-2 omicron variants in people with immunodeficiencies. Lancet Microbe. 4, E58–E59. doi: 10.1016/S2666-5247(22)00297-X 36332646 PMC9625114

[B35] Oliver M. A. Meredith R. T. Smith B. R. Bermingham M. D. Brackett N. F. Chapman M. D. (2022). Correction: longitudinal T cell responses against ancestral, delta, and omicron sars-Cov-2 variants determined by rapid cytokine release assay in whole blood. Immunohorizons 6, 835–836. doi: 10.4049/immunohorizons.2200090 36547386

[B36] Osmanodja B. Ronicke S. Budde K. Jens A. Hammett C. Koch N.. (2022). Serological response to three, four and five doses of sars-Cov-2 vaccine in kidney transplant recipients. J. Clin. Med. 11, 2565. doi: 10.3390/jcm11092565 35566691 PMC9105533

[B37] Oyaert M. De Scheerder M. A. Van Herrewege S. Laureys G. Van Assche S. Cambron M.. (2022). Evaluation of humoral and cellular responses in sars-Cov-2 mrna vaccinated immunocompromised patients. Front. Immunol. 13, 858399. doi: 10.3389/fimmu.2022.858399 35401575 PMC8988283

[B38] Parker E. P. K. Desai S. Marti M. Nohynek H. Kaslow D. C. Kochhar S.. (2022). Response to additional covid-19 vaccine doses in people who are immunocompromised: a rapid review. Lancet Glob Health 10, e326–e328. doi: 10.1016/S2214-109X(21)00593-3 35180408 PMC8846615

[B39] Peghin M. Graziano E. Grossi P. A. (2022). Sars-Cov-2 vaccination in solid-organ transplant recipients. Vaccines (Basel) 10, 1430. doi: 10.3390/vaccines10091430 36146506 PMC9503203

[B40] Petrone L. Petruccioli E. Vanini V. Cuzzi G. Najafi Fard S. Alonzi T.. (2021). A whole blood test to measure sars-Cov-2-Specific response in covid-19 patients. Clin. Microbiol. Infect. 27, 286 E7–286 E13. doi: 10.1016/j.cmi.2020.09.051 PMC7547312 33045370

[B41] Prendecki M. Thomson T. Clarke C. L. Martin P. Gleeson S. De Aguiar R. C.. (2021). Immunological responses to sars-Cov-2 vaccines in kidney transplant recipients. Lancet 398, 1482–1484. doi: 10.1016/S0140-6736(21)02096-1 34619100 PMC8489877

[B42] Reischig T. Kacer M. Vlas T. Drenko P. Kielberger L. Machova J.. (2022). Insufficient response to mrna sars-Cov-2 vaccine and high incidence of severe covid-19 in kidney transplant recipients during pandemic. Am. J. Transplant. 22, 801–812. doi: 10.1111/ajt.16902 34860470 PMC9906453

[B43] Rincon-Arevalo H. Choi M. Stefanski A. L. Halleck F. Weber U. Szelinski F.. (2021). Impaired humoral immunity to sars-Cov-2 Bnt162b2 vaccine in kidney transplant recipients and dialysis patients. Sci. Immunol. 6, eabj1031. doi: 10.1126/sciimmunol.abj1031 34131023

[B44] Saleem B. Ross R. L. Bissell L. A. Aslam A. Mankia K. Duquenne L.. (2022). Effectiveness of sars-Cov-2 vaccination in patients with rheumatoid arthritis (Ra) on dmards: as determined by antibody and T cell responses. Rmd Open 8, e002050. doi: 10.1136/rmdopen-2021-002050 35365569 PMC8977455

[B45] Schuurman J. Perdok G. J. Lourens T. E. Parren P. W. Chapman M. D. Aalberse R. C. (1997). Production of a Mouse/Human chimeric ige monoclonal antibody to the house dust mite allergen der p 2 and its use for the absolute quantification of allergen-specific ige. J. Allergy Clin. Immunol. 99, 545–550. doi: 10.1016/S0091-6749(97)70083-6 9111501

[B46] Simader E. Tobudic S. Mandl P. Haslacher H. Perkmann T. Nothnagl T.. (2022). Importance of the second sars-Cov-2 vaccination dose for achieving serological response in patients with rheumatoid arthritis and seronegative spondyloarthritis. Ann. Rheum Dis. 81, 416–421. doi: 10.1136/annrheumdis-2021-221347 34844927

[B47] Simon D. Tascilar K. Fagni F. Schmidt K. Kronke G. Kleyer A.. (2022). Efficacy and safety of sars-Cov-2 revaccination in non-responders with immune-mediated inflammatory disease. Ann. Rheum Dis. 81, 1023–1027. doi: 10.1136/annrheumdis-2021-221554 34819271

[B48] Smith R. M. Cooper D. J. Doffinger R. Stacey H. Al-Mohammad A. Goodfellow I.. (2022). Sars-Cov-2 vaccine responses in renal patient populations. BMC Nephrol. 23, 199. doi: 10.1186/s12882-022-02792-w 35641961 PMC9153874

[B49] Stumpf J. Siepmann T. Lindner T. Karger C. Schwobel J. Anders L.. (2021). Humoral and cellular immunity to sars-Cov-2 vaccination in renal transplant versus dialysis patients: a prospective, multicenter observational study using mrna-1273 or Bnt162b2 mrna vaccine. Lancet Reg. Health Eur. 9, 100178. doi: 10.1016/j.lanepe.2021.100178 34318288 PMC8299287

[B50] Suthar M. S. Zimmerman M. G. Kauffman R. C. Mantus G. Linderman S. L. Hudson W. H.. (2020). Rapid generation of neutralizing antibody responses in covid-19 patients. Cell Rep. Med. 1, 100040. doi: 10.1016/j.xcrm.2020.100040 32835303 PMC7276302

[B51] Tan A. T. Lim J. M. Le Bert N. Kunasegaran K. Chia A. Qui M. D.. (2021). Rapid measurement of sars-Cov-2 spike T cells in whole blood from vaccinated and naturally infected individuals. J. Clin. Invest. 131, e152379. doi: 10.1172/JCI152379 34623327 PMC8409582

[B52] Tang P. C. H. Ng W. H. King N. J. C. Mahalingam S. (2022). Can live-attenuated sars-Cov-2 vaccine contribute to stopping the pandemic? PloS Pathog. 18, E1010821. doi: 10.1371/journal.ppat.1010821 36129963 PMC9491521

[B53] Tortellini E. Zingaropoli M. A. Mancarella G. Marocco R. Carraro A. Jamhour M.. (2022). Quality of T-cell response to sars-Cov-2 mrna vaccine in art-treated plwh. Int. J. Mol. Sci. 23, 14988. doi: 10.3390/ijms232314988 36499317 PMC9741180

[B54] Trimpert J. Adler J. M. Eschke K. Abdelgawad A. Firsching T. C. Ebert N.. (2021). Live attenuated virus vaccine protects against sars-Cov-2 variants of concern B.1.1.7 (Alpha) and B.1.351 (Beta). Sci. Adv. 7, Eabk0172. doi: 10.1126/sciadv.abk0172 34851677 PMC8635430

[B55] Watson O. J. Barnsley G. Toor J. Hogan A. B. Winskill P. Ghani A. C. (2022). Global impact of the first year of covid-19 vaccination: a mathematical modelling study. Lancet Infect. Dis. 22, 1293–1302. doi: 10.1016/S1473-3099(22)00320-6 35753318 PMC9225255

[B56] Xie Y. Liu Y. Liu Y. (2022). The flare of rheumatic disease after sars-Cov-2 vaccination: a review. Front. Immunol. 13, 919979. doi: 10.3389/fimmu.2022.919979 35860285 PMC9289284

[B57] Xu X. Vesterbacka J. Aleman S. Nowak P. Group C. S. (2022). High seroconversion rate after vaccination with mrna Bnt162b2 vaccine against sars-Cov-2 among people with hiv - but hiv viremia matters? Aids 36, 479–481. doi: 10.1097/QAD.0000000000003135 35084386

[B58] Zhang X. Wu S. Wu B. Yang Q. Chen A. Li Y.. (2021). Sars-Cov-2 omicron strain exhibits potent capabilities for immune evasion and viral entrance. Signal Transduction And Targeted Ther. 6, 430. doi: 10.1038/s41392-021-00852-5 PMC8678971 34921135

