# Supplementary material for: Differential cellular and humoral immune responses in immunocompromised individuals following multiple SARS-CoV-2 vaccinations
Source: Front Cell Infect Microbiol. 2023 Jun 23;13:1207313. doi: 10.3389/fcimb.2023.1207313 (PMC10327606; doi:10.3389/fcimb.2023.1207313)
Supplement: Supplementary file 1 [file DataSheet_1.pdf]

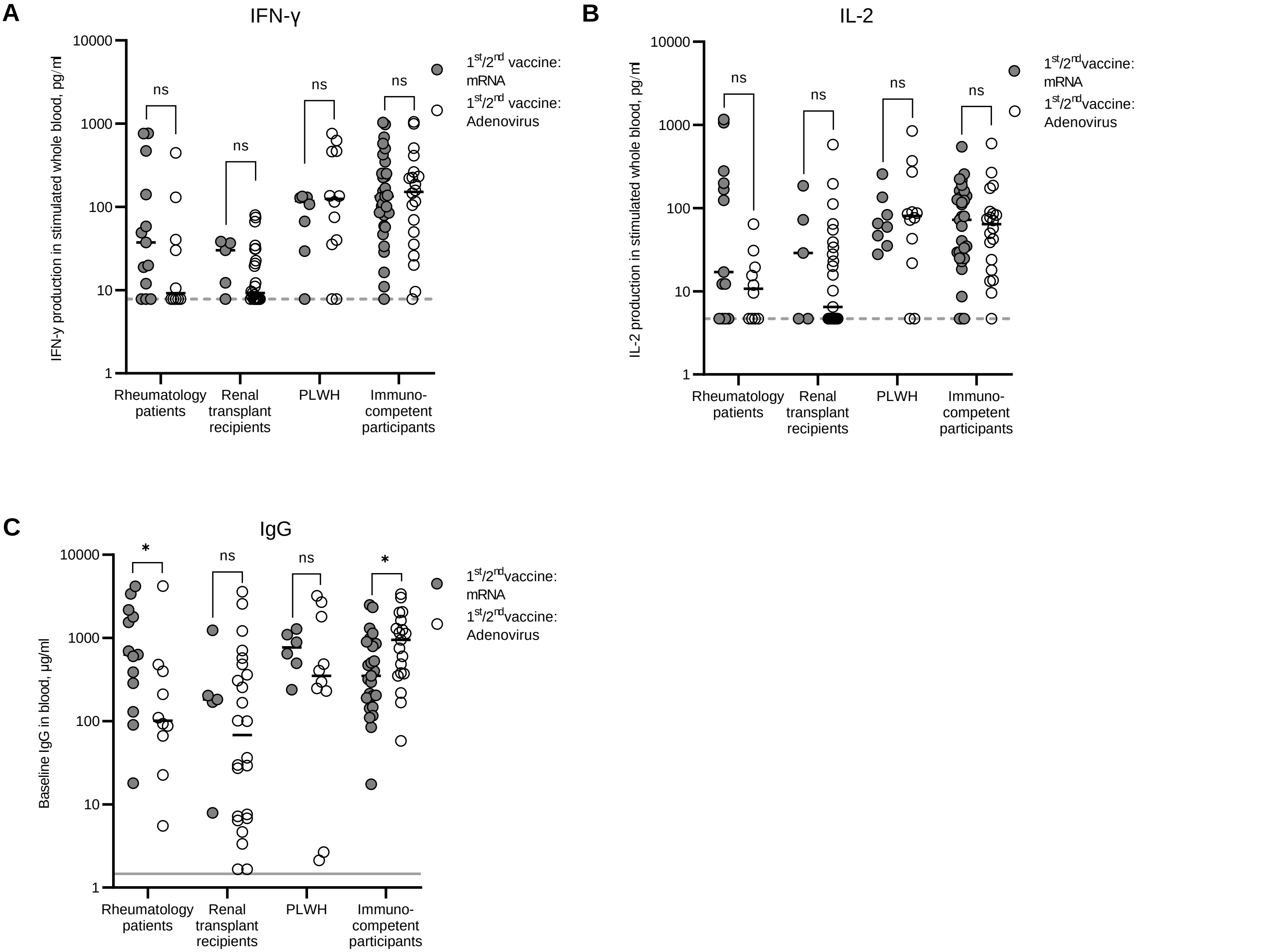

Supplemental Figure S1. Immune responses in immunocompromised and immunocompetent cohorts according to initial SARS-COV-2 vaccine type

IFN-γ (**A**) and IL-2 (**B**) production in whole blood in response to overnight peptide stimulation and baseline measurement of spike-specific IgG antibodies (**C**) in immunocompromised and immunocompetent cohorts. ● = mRNA as initial vaccine doses, ○ = adenovirus vaccine as initial vaccine doses. Results show individual results with median. Statistical analysis was performed with a Mann Whitney test. *p<0.05, ns = not significant. Dotted line indicates lower limit of detection. Solid grey line indicates the mean IgG concentration of three pre-pandemic plasma samples.

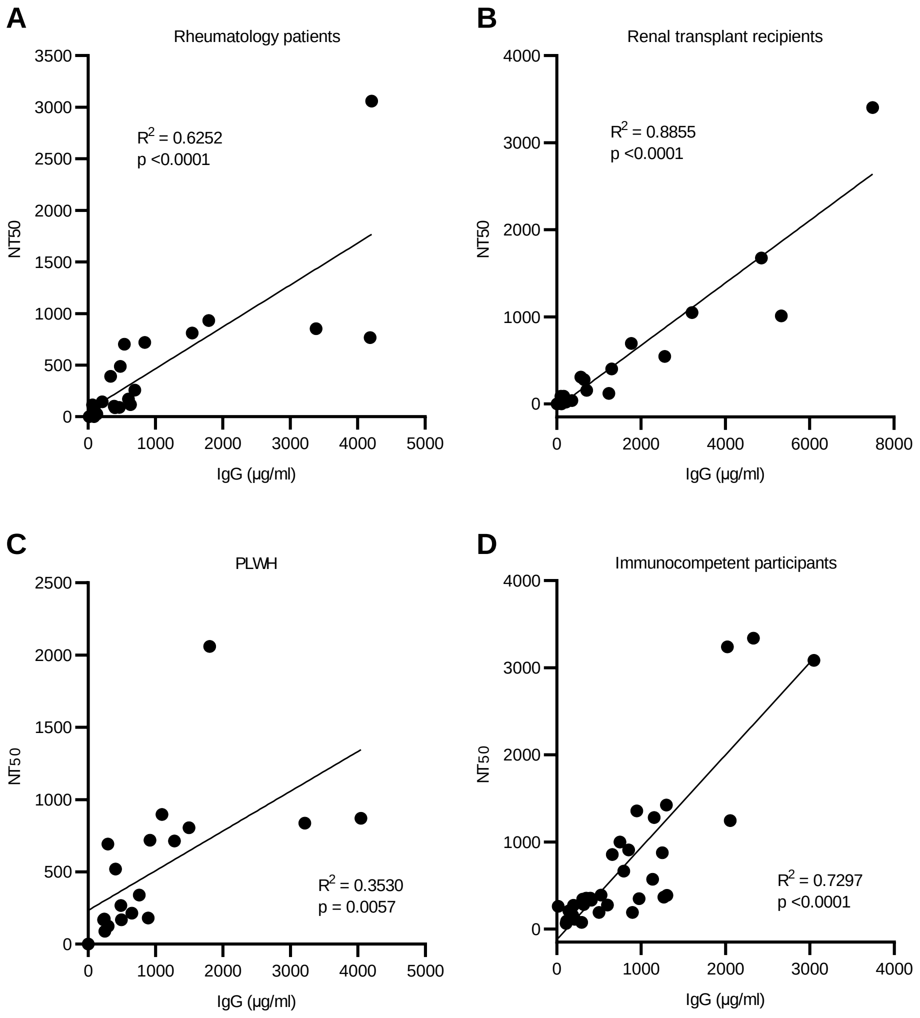

Supplemental Figure S2. Correlation of results from the 50% neutralising titre (NT50) assay with the spike-specific IgG antibody assay

Regression analysis of NT50 with IgG (µg/ml) in rheumatology patients (**A**), renal transplant recipients (**B**), PLWH (**C**) and immunocompetent participants (**D**). Statistical analysis was performed by simple regression analysis. R squared and p values are indicated, each symbol represents an individual donor.

Supplemental Table 1. Participant information for rheumatology patients

Underlying rheumatology condition	Current biologic
Ankylosing spondylitis = 5	Abatacept = 4
Axial Spondyloarthropathy = 1	Adalimumab = 2
Chronic plaque psoriasis = 1	Benepali = 3
Erosive rheumatoid arthritis =1	Cyclophosphamide = 1
Fibroblastic rheumatoid arthritis = 1	Golimumab = 1
Juvenile idiopathic arthritis (JIA) = 1	Inflectra/Infliximab = 14
Multi systematic sarcoidosis = 1	TCZ = 4
Nonspecific connective tissue disorder/ILD = 1	
Psoriatic arthritis = 1	
Rheumatoid arthritis = 12	
Seronegative Rheumatoid Arthritis = 4	

Supplemental Table 2. Participant information for renal transplant recipients

Underlying renal diagnosis	Current immunosuppressive drugs
Glomerular diseases = 16	Tacrolimus and mycophenolate mofetil = 19
IgA nephropathy = 7	Tacrolimus and prednisolone = 8
Focal segmental glomerulosclerosis (FSGS) = 2	Tacrolimus, mycophenolate mofetil and prednisolone = 19
Membranous nephropathy = 1	
Membranoproliferative glomerulonephritis = 1	
Anti-neutrophil cytoplasmic antibody (ANCA)-associated vasculitis = 1	
Otherwise unspecified chronic glomerulonephritis = 4	
Diabetic nephropathy = 6	
Polycystic kidney disease = 6	
Primary reflux nephropathy (sporadic) = 2	
Chronic pyelonephritis = 2	
Hypertensive nephropathy = 2	
Chronic interstitial nephritis = 1	
Obstructive uropathy = 1	
Tubulointerstitial nephritis = 1	
Alport syndrome = 1	
Unknown = 8	

Supplemental Table 3. Regression analysis of IFN-γ, IL-2 and IgG with age. R-squared and p values are shown. Result in ***bold*** indicates statistical significance ($p < 0.05$)

Group	IFN-γ R-squared	IFN-γ p value	IL-2 R-squared	IL-2 p value	IgG R-squared	IgG p value
Rheumatology patients	0.0686	0.1698	0.0559	0.2167	0.0021	0.8137
Renal transplant recipients	0.0319	0.2348	0.0676	0.0809	0.0084	0.5496
PLWH	0.0756	0.1652	0.0175	0.5102	0.0040	0.7702
Immunocompetent participants	0.0854	***0.0338***	0.0045	0.6333	0.0570	0.1187

Supplemental Table 4. Mann-Whitney test comparing IFN-γ, IL-2 and IgG results in males and females. p values are shown. Result in ***bold*** indicates statistical significance ($p < 0.05$)

Group	IFN-γ **p value**	IL-2 p value	IgG p value
Rheumatology patients	0.7982	0.8072	*0.0457*
Renal transplant recipients	0.7055	0.7857	0.6827
PLWH	0.9332	0.2670	0.3282
Immunocompetent participants	0.1484	0.0549	0.6651
